# Microbial Programming of Systemic Innate Immunity and Resistance to Infection

**DOI:** 10.1371/journal.ppat.1004506

**Published:** 2014-12-04

**Authors:** Thomas B. Clarke

**Affiliations:** MRC Centre for Molecular Bacteriology and Infection, Imperial College London, London, United Kingdom; University of Notre Dame, United States of America

## Introduction

A multicellular organism is subject to constant microbial exposure throughout its life. These interactions can be transient or permanent, with microbes ranging from those considered commensal to pathogenic [1]. The initial host response to these myriad microbes relies upon the innate immune system. This highly conserved and ancient arm of host defense has generally been thought of as hardwired and inflexible [2], with only local interactions with microbes at environmental interfaces believed to exert a significant influence on innate immune cell function [3], [4]. It is now becoming clear that this long-held dogma is incorrect, and the ongoing microbial exposure we experience throughout life, whether it is by the microbiota or pathogenic organisms, exerts a systemic influence on the production and function of innate immune cells. In this Pearl, I will describe the mechanistic basis for these systemic effects and discuss how they modulate host defenses to infection by other microbes.

## Systemic Regulation of Macrophage, Neutrophil, and Dendritic Cell Function by the Microbiota Enhances Innate Defenses against Infection

**Figure 1:**
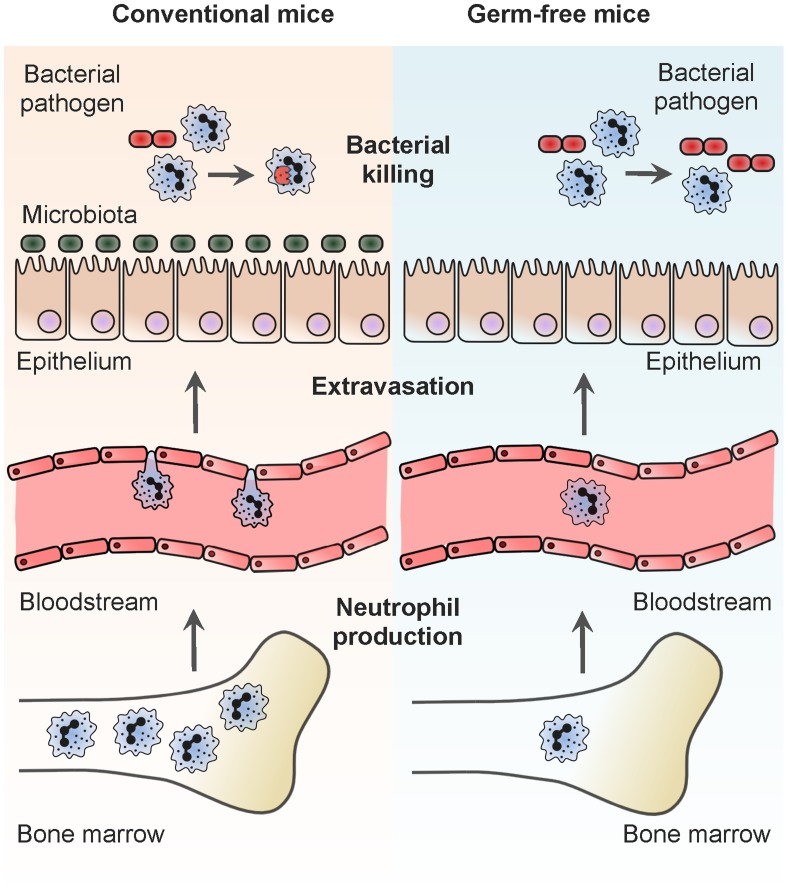
Systemic regulation of neutrophil production and function by the microbiota. This summarizes the stages in neutrophil production and function that are known to be regulated by the microbiota. Conventional mice are those colonized by the microbiota and germ-free mice are those that have been born and raised in a sterile environment and thus not colonized by any live microbial communities. Neutrophil production: the site of neutrophil production is the bone marrow, and signals from the microbiota, which can be mediated via TLR4, stimulate neutrophil production continually under steady-state conditions. Neutrophil extravasation: to protect against infection, neutrophils are recruited from the bloodstream to inflamed tissues. In the absence of TLR stimulation from the microbiota, the ability of neutrophils to navigate this migratory step, in response to microbial signals, is diminished. Neutrophil killing of bacteria: neutrophils leave the bone marrow fully differentiated and mature. While in the bone marrow, bacterial cell wall peptidoglycan is recognized by Nod1, primes the antibacterial capacity of neutrophils, and facilitates more effective killing of bacterial pathogens at sites of recruitment.

Tissue resident macrophages and dendritic cells (DCs), combined with recruited neutrophils, are major innate effector cells that form the first line of host defense to protect against infection and help maintain tissue homeostasis. The impact of microbes on the production and functional programming of macrophages and dendritic cells has generally been assumed limited to the mucosa, with colonizing microbes known to fine-tune the function of these cells at this site. Microbial influences on neutrophils have been thought restricted to severe infections, where there can be a short-term increase in neutrophil production, but neutrophil function has been believed to be subject to minimal microbial influence because of their terminal differentiation and short half-life [5]. Recent work has brought about a reevaluation of these views and has shown that neutrophils are actually subject to microbial regulation throughout their life even in the absence of infection (Fig. 1). This starts with their production, as mice devoid of any live microbial communities (germ-free) produce fewer neutrophils, compared to conventional mice colonized by the microbiota [6]. Functionally, circulating neutrophils in germ-free mice have defects in extravasation from the bloodstream into target tissues in response to microbial signals [7] and also in killing of bacterial pathogens [8]. Thus, signals from the microbiota have a systemic effect on neutrophils promoting their production and antimicrobial capacity. The role of neutrophils has now been shown to extend beyond this acute innate response into the regulation of adaptive immunity. Somatic hypermutation and antibody production by B-cells in the spleen is, in part, controlled by splenic neutrophils and, analogously to the innate function of neutrophils, this novel aspect of neutrophil biology is also thought to be promoted by the microbiota [9]. Like neutrophils, macrophage populations in systemic, nonmucosal tissues are also subject to microbial regulation. In the absence of the microbiota, splenic macrophage numbers are reduced, as are the expression of host defense genes, including those encoding proteins involved in antiviral immunity, such as type I interferon [10], [11]. In another example of the long-range influence the microbiota can have on macrophage function, it has been shown that signals from intestinal bacteria promote reactive oxygen species (ROS) production by alveolar macrophages in the lung in response to bacterial pathogens [12]. In the absence of this stimulation, ROS production during infection is reduced, resulting in attenuated early clearance of bacteria from the lung [12]. Collectively, this suggests that with reduced microbial burden, the host is economical with its resources, diverting fewer to innate cell production and minimizing the production of costly molecules that could lead to tissue damage, such as inflammatory cytokines and ROS. In contrast to neutrophils and macrophages, the number of dendritic cells in nonmucosal tissues is thought to be equivalent between germ-free and conventional mice, suggesting that the microbiota does not regulate dendritic cell production systemically [13]. The microbiota does, however, play a significant role in shaping their function, as splenic dendritic cells isolated from germ-free mice and then stimulated with PRR ligands, express significantly less *il6*, *tnfa*, *il12*, *il18*, and type I interferon, in comparison to the same cells from conventionalized animals [13]. The proposed mechanistic basis for this is via epigenetic modification of the promoters of the genes encoding these proinflammatory cytokines. Splenic DC isolated from conventional mice have greater trimethylation of histone 3 lysine 4 (H3K4), compared to the same cells from germ-free animals [13], which is characteristic of genes undergoing active transcription, and therefore microbiota-dependent chromatin modifications could be the basis for the increased sensitivity of dendritic cells from conventional mice to microbial stimulation. Further functional defects in dendritic cells have been identified in other work, showing that the migration of dendritic cells from the lung to mediastinal lymph nodes during influenza infection is severely impaired in the absence of the microbiota [14]. Emerging from these data is a clear picture showing that the microbiota not only has a proximal influence on innate cells at the mucosa but also has a distal influence, regulating the function of systemic innate cells during homeostasis. This regulation of innate cell function by bacteria within the microbiota leads to enhanced innate defense to infection by viruses and bacteria. Microbiota-dependent stimulation of neutrophils enhances host defenses against systemic bacterial infection while it has been shown that systemic programming of macrophage and dendritic cells function provides more robust antibacterial and antiviral immunity in the lung [8], [10]–[14].

## Pattern Recognition Establishes the Level of Innate Immune Activation

A common theme apparent from this work is that the mechanistic basis for many of these systemic phenomena involves the activation of pattern recognition receptors (PRRs), host receptors that recognize conserved microbial structures found in all microbes, whether they are considered pathogens or nonpathogens. Steady-state neutrophil production is regulated by Toll-like receptor 4 (Tlr4) activation [6], extravasation is enhanced in response to redundant Toll-like receptor (TLR) activation via MyD88 [7], and bacterial killing is promoted by recognition of bacterial cell wall peptidoglycan by the Nod-like receptor (NLR) 1, Nod1 (Fig. 1) [8]. Similarly, PRR activation can drive the systemic activation of macrophages and dendritic cells, for example, enhanced ROS production by alveolar macrophages is promoted by NLR ligands originating from the intestine [12] and impaired dendritic cell migration during influenza infection in the absence of the microbiota can be rescued by intrarectal administration of lipopolysaccharide (LPS) [14]. But how do microbes within the microbiota and their products at the mucosa exert a systemic effect on innate cells in distal tissues via PRRs? Recent work shows that the boundary between the external microbial environment delimited by the mucosa, and sterile, internal, nonmucosal tissues is more blurred than previously recognized. Multiple studies [8], [9], [15], [16] and our unpublished data (Clarke, T.B. unpublished) have found microbial products throughout the body, and these disseminated products are known to exert some of the systemic effects of the microbiota. For example, peptidoglycan recognized by Nod1 and required for systemic priming of neutrophil function is found in multiple host tissues and accumulates in the bone marrow, where priming takes place [8]. Furthermore, neutrophils in the spleen have been shown to contain bacterial DNA and LPS [9], providing further evidence for the continual dissemination of bacterial products systemically. Another, nonmutually exclusive mechanism can be through local PRR activation by the microbiota at environmental interfaces leading to the production of host signaling molecules that can exert systemic effects on systemic innate cells. TLR signaling resulting in mucosal IL-17A production has been shown to be important for promoting neutrophil granulocytosis in neonatal mice [17] and provides an example of this second mechanism. Taken together, these studies are beginning to show that PRRs act as homeostatic regulators of the systemic immune system and gauge the required level of innate cell activity through continual tonic engagement by microbial products.

## Programming of Innate Immunity Is Not Limited to the Microbiota

In the examples outlined above, programming of innate cell function was achieved by microbes and microbial products from the microbiota. However, these are not the only microbes we interact with, and this, therefore, raises the question of whether microbial modulation of innate immunity is somehow unique to the microbiota or can also be mediated by other microbial encounters. For humans, these interactions can be infection with pathogenic microbes or vaccination. Vaccination with Bacillus Calmette-Guérin (BCG), an attenuated strain of *Mycobacterium bovis*, protects against tuberculosis (TB), but its effects on the immune system extend far beyond specific protection against TB [18]. BCG vaccination has been shown to afford nonspecific protection against infection by a number of pathogens, including *Schistosoma mansoni* and *Listeria monocytogenes* [18]. This is, in part, mediated by programming of circulating monocytes, macrophage, and dendritic cell precursors that are also potent innate cells prior to their differentiation [19]. BCG vaccination increases the expression of activation markers CD14 and CD11b on circulating monocytes, and upon stimulation of these cells by PRR ligands or bacteria, they produce significantly more IL-1β and TNFα than the same cells from unvaccinated individuals [19]. Using mouse models, these effects were shown to be independent of the adaptive immune system and required PRR activation, specifically, the NLR Nod2 [19]. Again, in line with the effects of the microbiota on innate cells, the promoters of genes showing increased production upon stimulation had greater levels of trimethylation of H3K4 [19].

The ability of one infection to influence early host responses to infection by another, unrelated pathogen has also been known for a long time. The mechanistic basis for this has been previously ascribed to heterologous immunity [20], a phenomena whereby the memory lymphocytes that developed in response to one pathogen recognize, and are activated by, a crossreactive antigenic epitope present on another unrelated pathogen [21]. In addition to these effects on adaptive immunity, infection-induced changes in innate cell function have now been shown to shape host defense systemically. Intranasal infection with herpesvirus promotes macrophage production of type II interferon, and this helps protect against systemic bacterial infection, independently of any component of adaptive immunity [22]. However, chronic immune activation because of infection or excessive microbial stimulation can also result in defects in immune function [23]. These detrimental effects have been most comprehensively studied from the perspective of the adaptive immune system, with comparatively less known of the systemic effects of chronic infection on innate cells and how this influences subsequent infection [23]. However, it is well established that the extreme microbial stimulation that occurs during sepsis causes lasting alterations in innate cell function, including reduced ROS production by neutrophils in the bone marrow that can lead to poorer clearance of bacterial lung infection [24]. Additionally, it is known that patients with concurrent intestinal helminth and TB infection have reduced natural killer cell numbers in comparison to those with TB alone [25]. Thus, more information is required to understand the beneficial and detrimental effects different microbial groups can have on innate immunity.

## Conclusion

It is clear from this work that innate immunity is a far more flexible and responsive system than previously appreciated. The innate immune system is constantly responding to its surroundings and adjusting the production and function of innate effector cells to match the microbial challenge of its current environment. This is not limited to the mucosa but is also true of the systemic immune compartment, showing the profound influence microbes have on shaping the entire immune system. This fundamental microbial programming of innate immunity, often through PRR signaling, allows economical use of resources, avoiding unnecessary production of potentially tissue damaging molecules like ROS, while gauging the required level of immune activation to effectively deal with infection. Microbial programming of innate immunity resulting in enhanced protection against infection can be mediated by myriad microbes, ranging from members of the microbiota to pathogens, suggesting that we need to move beyond this simplistic dichotomy to fully comprehend the microbial groups that regulate different aspects of immune function. It might then be possible to harness the power of specific microbial groups to exploit the flexibility to the innate immune system for therapeutic benefit to improve responses to vaccination and help protect against infections.
